# Transposable element finder (TEF): finding active transposable elements from next generation sequencing data

**DOI:** 10.1186/s12859-022-05011-3

**Published:** 2022-11-22

**Authors:** Akio Miyao, Utako Yamanouchi

**Affiliations:** Institute of Crop Science, National Agriculture and Food Research Organization, 2-1-2, Kannondai, Tsukuba, Ibaraki 305-8518 Japan

**Keywords:** Transposable element, Retrotransposon, Next generation sequence, Target site duplication, *Tos17*

## Abstract

**Background:**

Detection of newly transposed events by transposable elements (TEs) from next generation sequence (NGS) data is difficult, due to their multiple distribution sites over the genome containing older TEs. The previously reported Transposon Insertion Finder (TIF) detects TE transpositions on the reference genome from NGS short reads using end sequences of target TE. TIF requires the sequence of target TE and is not able to detect transpositions for TEs with an unknown sequence.

**Result:**

The new algorithm Transposable Element Finder (TEF) enables the detection of TE transpositions, even for TEs with an unknown sequence. TEF is a finding tool of transposed TEs, in contrast to TIF as a detection tool of transposed sites for TEs with a known sequence. The transposition event is often accompanied with a target site duplication (TSD). Focusing on TSD, two algorithms to detect both ends of TE, TSDs and target sites are reported here. One is based on the grouping with TSDs and direct comparison of *k-*mers from NGS without similarity search. The other is based on the junction mapping of TE end sequence candidates. Both methods succeed to detect both ends and TSDs of known active TEs in several tests with rice, *Arabidopsis* and *Drosophila* data and discover several new TEs in new locations. PCR confirmed the detected transpositions of TEs in several test cases in rice.

**Conclusions:**

TEF detects transposed TEs with TSDs as a result of TE transposition, sequences of both ends and their inserted positions of transposed TEs by direct comparison of NGS data between two samples. Genotypes of transpositions are verified by counting of junctions of head and tail, and non-insertion sequences in NGS reads. TEF is easy to run and independent of any TE library, which makes it useful to detect insertions from unknown TEs bypassed by common TE annotation pipelines.

**Supplementary Information:**

The online version contains supplementary material available at 10.1186/s12859-022-05011-3.

## Background

TEs are mobile elements in the genome. In eukaryotes, TEs are one of the major components of the genome due to their self-propagation and transposition. TEs are categorized into two classes [1, 2]. Class I is RNA type retrotransposon. Class II is DNA type. Class I retrotransposon is transcribed to RNA, and then reverse-transcribed DNA by self-coding reverse-transcriptase is inserted into the genome sequence. The ‘copy-and-paste’ manner promotes the increase of the elements in the genome. Class II DNA type transposon shows ‘cut-and-paste’ manner. Most retrotransposons and DNA transposons make the TSD at the integration. Typical size of TSD is from 3 to 10 bases. The TSD is a footprint for events of transposition of TEs.

A lot of effort has been spent by biological and computational scientists to detect TE in the genome and transposition events of TE. For example, to find active retrotransposons, PCR amplification using primer pairs on conserved pol gene of rice was performed with reverse transcribed DNA. The specific amplification of pol gene in the callus of rice led to the detection of active retrotransposon *Tos17* [3]. Retrotransposon *Tos17* is only active in cultured cells of rice. In regenerated individuals from cultured cells, *Tos17s* are immediately inactivated and stay at transposed positions on the rice genome. Class II transposon, *mPing* was found in the *sl* gene from slender glume mutant of rice, also detected by the transposon display method [4, 5]. Most computer analyses to detect TE are based on similarity against the reference genome sequence or consensus sequences of TE, analogous to the experimental method of PCR with primer pairs from the conserved region [6].

Non-similarity-based approaches will detect the dynamics of TE from a different angle. When TE is transposed in an individual genome, the new NGS reads will appear containing 5’(head) and 3’(tail) TE sequences. Insertion positions can then be identified by the NGS pair containing same TSD sequence flanking head and tail sequences. Previously, the TE mapping program, Transposon Insertion Finder (TIF), implemented with the TSD pairing algorithm, has been reported [7].

If TSDs, as a result of TE transposition, are present in the NGS reads, an active TE will be found by associating TSD with flanking head and tail sequence of TE. In this paper, two algorithms to detect head and tail sequences of a transposed TE are presented. The algorithms have been implemented as a perl program named ‘Transposable Element Finder’ (TEF). The TEF program works by detecting the head and tail sequences of transposed TEs.

## Results

### Algorithms

**Fig. 1:**
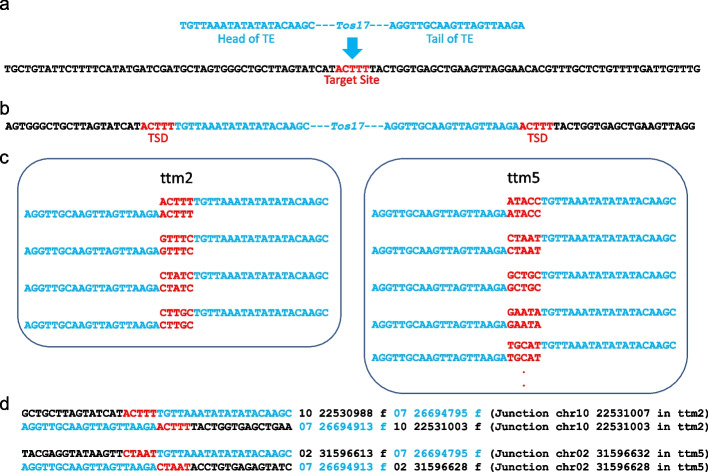
Transposition of retrotransposon *Tos17* and new sequences in NGS short reads due to the transposition. **a** *Tos17* indicated with head and tail sequences (20 bases in blue) is transposed into the target site (red) of genome sequence (black). **b** After the integration of *Tos17*, five base of target site is duplicated at ends of the head and tail sequences. **c** Because *Tos17* was inserted into different positions of genome in ttm2 and ttm5, TSD sequences flanking the head and tail sequence of *Tos17* should be different between ttm2 and ttm5. **d** Detection of junctions in 40 base sequences. Values following sequence are chromosome, position, and direction of first 20 base, and chromosome, position, and direction of last 20 base. **TSD method:** 25 base (20 base head or tail plus TSD length) sequences are sliced from fastq short reads in each position. Sliced 25 base sequences are paired with same TSD sequence (red) and the grouped with head and tail sequences (blue). If the types of TSD are different between ttm2 and ttm5, the head and tail sequence pair must be ends of TE (panel **c**). **Junction method:** 20 base length flanking sequences (black) including TSD (red) and adjacent 20 base length candidates of TE head or tail sequence in panel **b** are mapped to reference genome sequence (panel **d**). Map positions of first and adjacent 20 base sequences of all sliced 40 base sequences from fastq short reads are obtained. If adjacent 20 base (blue) is mapped to different position on the reference genome sequence, the adjacent sequence may be a head or tail sequence of TE. Select head and tail pair mapped by flanking sequences to different positions on the reference sequence within and between ttm2 and ttm5. The position of head junction in ttm2 is 22530988 + 20 − 1 = 22531007

Both algorithms are focused on TSDs created at transpositions and require whole genome sequence data from two samples of A and B. In the case of TE transposed into a different genome position between sample A and B, TEF algorithms detect head, tail and TSD sequences of TE candidates. Algorithm 1 (TSD method) selects 20 bases long head and tail sequences of TE candidate accompanied by same TSD sequence. Algorithm 2 (Junction method) selects head and tail pair of TE candidate whose flanking sequences are mapped on nearby (equaling TSD length) positions in the reference genome. Figure 1 shows the principle of the algorithms using the same data as described in the TIF paper. For example, retrotransposon *Tos17* (shown in blue) is inserted into a target site (shown in red) of genome (Fig. 1a). After the integration, five bases of target site are duplicated, adjacent to head and tail sequences of TE (Fig. 1b).

For the algorithm 1 (TSD method), if the TE is transposed in regenerated individuals (ttm2 and ttm5), head and tail pair of TE with different combinations of TSD sequence must be found in NGS sequence (Fig. 1c). The TIF makes paired flanking sequences (shown in black, Fig. 1b) of TE head and tail with same TSD sequence to detect transposed positions on the genome. In contrast to TIF, TEF makes pairs from TE (shown in blue, Fig. 1c) head and tail candidates with same TSD sequence, and then TEF selects pairs of head and tail candidates with different kinds of TSDs between ttm2 and ttm5.

Algorithm 2 (Junction method) is based on detection of junction positions by mapping on reference genome sequence. Some fragments of *k-*mer (*k* = 40) from each sequence position of short reads must contain junction of transposed TE. Fragments from sequence of Fig. 1b and 1c containing the junctions are shown in Fig. 1d. If the first 20-mer is the upstream flanking sequence of TE head, following 20-mer will be mapped at different location of reference genome sequence, *i.e.,* original TE position before transposition. If the first 20-mer is TE tail, following 20-mer is downstream flanking sequence. From short-read sequences containing the junction of TE transposition, TE junctions from a pair of 40-mer sequence should be mapped on nearby (equal TSD length) positions and TE head and tail sequences are mapped on different locations of reference genome. The algorithm 2 searches pairs of flanking sequences which accompany same TSD, and the sequences are mapped at close positions. Head and tail sequence of TE corresponding the flanking sequences are selected.

## Algorithm 1 (TSD method)

1. For grouping head, tail and TSD sequences, cut out short reads derived from two samples A and B with a size of 20 bases + size of specified TSD while shifting each base by 1 base.
2. For their grouping, sort the cut-out sequences and output it together with the frequency.
3. Select A-specific and B-specific sequences, excluding sequences in both A and B. Common sequences between A and B do not contain the junction of transposed TEs.
4. From the selected A-specific and B-specific sequences, the sequences that can be candidates as TSD-Head and Tail-TSD sequences are output, respectively.
5. From the combination of TSD-Head and Tail-TSD sequences with same TSD, select a pair of Head and Tail sequences that have two or more TSD sequences.
6. Create a sorted file for A and B by outputting Head_Tail and TSD sequence on each line.
7. Select a Head_Tail pair in which the ratio of A-specific or B-specific TSD sequences to the total TSD sequences is greater than the threshold value (*e.g.*, > 0.7).
8. To count reads containing sites of transposition, select upstream section with head sequence (Upstream-Head) and downstream section with tail sequence (Tail-Downstream) in the fastq sequences from A and B.
9. Make non-insertion sequence, *i.e.,* wild-type sequence before TE transposition, by joining Upstream and Downstream sequences without a duplicated TSD sequence.
10. Count numbers of the Upstream-Head, Tail-Downstream, and non-insertion sequences in the fastq sequences from A and B.
11. Select Upstream-Head and Tail-Downstream sequences detected in only A or B, to eliminate already existing TEs.
12. If selected Upstream-Head and Tail-Downstream pair has corresponding non-insertion counts, the genotype of insertion should be heterozygous, otherwise, homozygous.
13. If a reference sequence is present, map the Upstream and Downstream sequences to determine the insertion site on the genome.

## Algorithm 2 (Junction method)

1. Cut out short reads derived from two samples A and B with a size of 40 bases while shifting each base by 1 base. If TE is transposed, the 40 base sequence must contain upstream flanking sequence (20 base length) and TE head sequence, and TE tail sequence and downstream flanking sequence (20 base length).
2. Sort the cut-out sequences and output them together with the frequency.
3. Select A-specific and B-specific sequences, excluding sequences in both A and B.
4. Exclude simple repeat sequences, if A, C, G or T is repeated ten times or more, or AC, AG, AT, TC or TG is repeated nine times or more in the first (head) or last (tail) 20 bases.
5. Get genomic position for both head and tail flanking sequences.
6. Sort by chromosome number and position.
7. Select head and tail pairs with TSD, and length of TSD is more than 2 and less than 17 bases.
8. Select head and tail pairs having multiple kinds of TSD sequence.
9. Select head and tail pairs with different kinds of TSDs between A and B.
10. Verify the transpositions by steps 8–13 of TSD method.

### Implementation of algorithms

Two algorithms have been implemented as a perl script tef.pl. Algorithm 1 (TSD method) requires fixed length of TSD. Head and tail sequences of TE candidate with same TSD sequence are selected. If the detected TSDs between sample A and B are different, the TE candidate of the head and tail sequence may be a part of active TE. Thus algorithm 1 detects active TE candidate without the reference genome sequence. On the other hand, algorithm 2 (Junction method) requires the reference genome sequence, because detection of map positions of flanking sequences with the head and tail are essential for algorithm 2. As flanking sequences mapped at closed positions on the reference genome are selected, algorithm 2 can detect ends of TE without specification of TSD length. Both algorithms examine all combinations of sliced *k-*mer from short reads. Parallel computing with multi-core CPU and multi process calculation is essential for detection of traces of the transposition in huge number of combinations. For TEF program, Multi-process style script was better than the multi-thread style within single perl process, due to the limit of the number of open files in one process and the memory handling features of perl. The summary of detected TEs is shown in Table 1.

**Table 1:** Summary of detected TEs

Species	Line	Accession	TEs by TEF	TEs by PCR	TEs from literature	Reference	New TEs found
Rice							
	ttm2	SRR556173	*Tos17*	*Tos17*	*Tos17*	3, 7, 8	
	ttm5	SRR556174 and SRR556175	*Tos17*	*Tos17*	*Tos17*	3, 7, 8	
	JRC01	DRR240814	*CACTA, Mutator, Dasheng, Tos5, Osr4.3, Osr25.1, Osr25.6, Osr26.7, Osr27.13, Osr27.31, Osr27.45, Osr27.55, Osr34, Ty3/gypsy,* Unclassified (29)	*Osr4.3*		8, 17, 18	*CACTA, Mutator, Dasheng, Tos5, Osr4.3, Osr25.1, Osr25.6, Osr26.7, Osr27.13, Osr27.31, Osr27.45, Osr27.55, Osr34, Ty3/gypsy,* Unclassified (29)
	JRC05	DRR240817	*CACTA, Mutator, Dasheng, Tos5, Osr4.3, Osr25.1, Osr25.6, Osr26.7, Osr27.13, Osr27.31, Osr27.45, Osr27.55, Osr34, Ty3/gypsy,* Unclassified (29)	*Tos5*		8, 17, 18	*CACTA, Mutator, Dasheng, Tos5, Osr4.3, Osr25.1, Osr25.6, Osr26.7, Osr27.13, Osr27.31, Osr27.45, Osr27.55, Osr34, Ty3/gypsy,* Unclassified (29)
	OsCmt3a-1	SRR1610772	*Tos17*, *Dasheng*, *Osr4.3*, *Osr25.4, Ty3/gypsy* (3)	nt	*Tos17, Dasheng, Osr4.3*	10	*Osr25.4, Ty3/gypsy*
	OsCmt3a-2	SRR1609962	*Tos17*, *Dasheng*, *Osr4.3*, *Osr25.4, Ty3/gypsy* (2)	nt	*Tos17, Dasheng, Osr4.3*	10	*Osr25.4, Ty3/gypsy*
*Arabidopsis*							
	ddm1(18H4)	DRR001193	*CACTA*, *Evade*, AT2G13940, AT5G44925, *Hi, MuDR-A, Ty1/copia* (2), *Ty3/gypsy* (1), Unclassified (10)	nt	*CACTA, Hi*	12, 14, 15	*Evade*, AT2G13940, AT5G44925, *MuDR-A, Ty1/copia (2), Ty3/gypsy (1),* Unclassified (11)
	ddm1(18J1)	DRR001194	*CACTA*, *Evade*, AT2G13940, AT5G44925, *Hi, Ty1/copia* (3), *Ty3/gypsy* (2), Unclassified (9)	nt	*CACTA, Hi*	12, 14, 15	*Evade*, AT2G13940, AT5G44925, *Ty1/copia* (3), *Ty3/gypsy* (2)*,* Unclassified (10)
	hc4	hc4	*ONSEN*	nt	*ONSEN*	9	
	hc31	hc31	*ONSEN*	nt	*ONSEN*	9	
*Drosophila*							
	RIL46	SRR82377	*P*, *FB*, *hobo*, *roo,* 412, *Diver, mgd1, hopper, Ty1/copi*a (4), *Ty3/gypsy* (2), Unclassified (4)	nt		7, 19, 20, 21	*P*, *FB*, *hobo*, *roo,* 412, *Diver, mgd1, hopper, Ty1/copi*a (4), *Ty3/gypsy* (2), Unclassified (4)
	RIL80	SRR82382	*P*, *FB*, *hobo*, *roo,* 412, *Diver, mgd1, mgd3, Ty1/copi*a (5), *Ty3/gypsy* (2), Unclassified (4)	nt		7, 19, 20, 21	*P*, *FB*, *hobo*, *roo,* 412, *Diver, mgd1, mgd3, Ty1/copi*a (5), *Ty3/gypsy* (2), Unclassified (4)
	33–27	SRR866314	*P, hobo, roo, Ty3/gypsy*	nt	*P, hobo, roo, 1360, Flea, Rt1, mdg*	19, 21, 24	*Ty3/gypsy*
	33–45	SRR866312	*P, hobo, hopper, roo, Ty3/gypsy*	nt	*P, hobo, roo, 1360, Flea, Rt1, mdg, G, hopper*	19, 21, 24	*Ty3/gypsy*

Numbers in brackets are detected number of TEs. For the JRC lines, only one sample for target TE was PCRed.

Nt Not tested. *P* eletemt was detected by only TSD method, because of absence of *P* element seqeunce in the reference genome. Details of detected TE are indicated in Additional file tables

### Detection of *Tos17* transposition

**Fig. 2:**
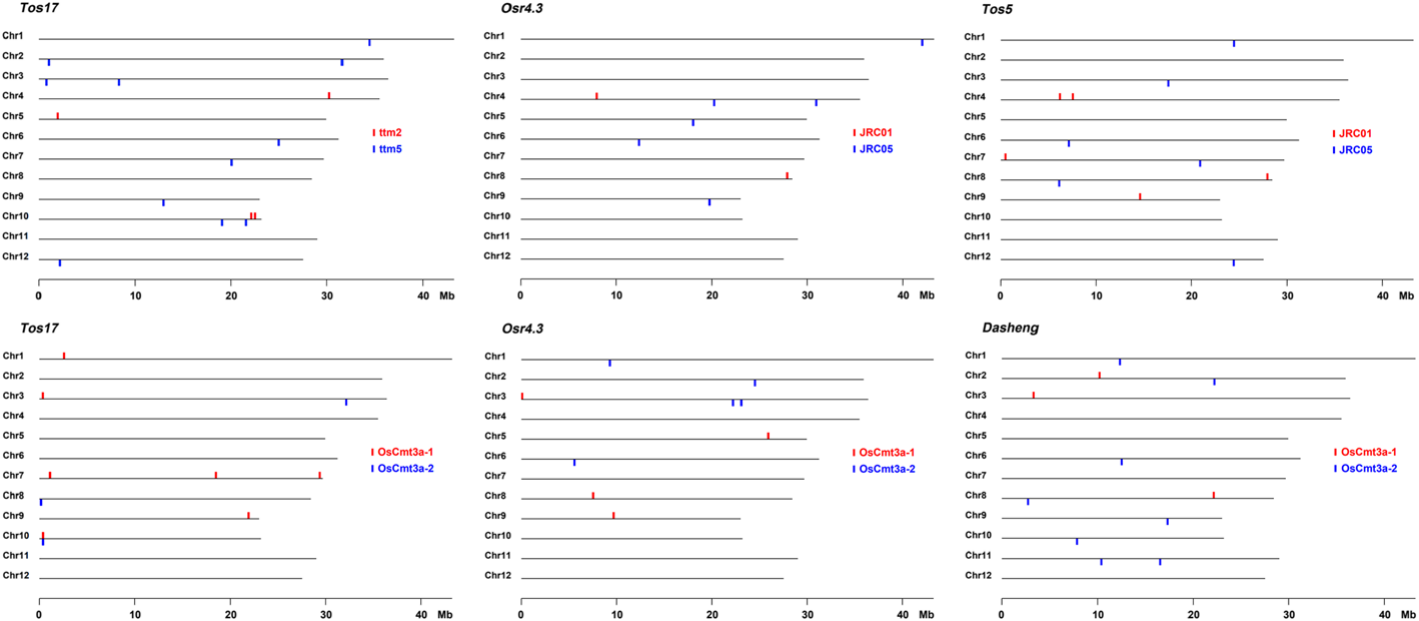
TE transpositions in rice. Transposition sites are indicated at top or bottom of each chromosome indicated by red or blue with line names. Lines ttm2 and ttm5 are regenerated individuals of rice. JRC01 and JRC05 are cultivars of rice core collection. Only transpositions of *Tos17* are detected in regenerated lines. On the other hand, transposition events of *Osr4.3 and Tos5* are detected in both JRC01 and JRC05. *OsCmt3a-1* and *OsCmt3a-2* are *Tos17* insertion mutants of chromomethylase gene in chromosome 10. Insertion positions of *Tos17* in *OsCmt3a* mutant are due to the activation and transposition of *Tos17* by the cell culture. *Tos17* did not activated in the chromomethylase mutant, and some insertions have been segregated out except homozygous insertions in chromosome 10. *Osr4.3* and *Dasheng* are activated and transposed in chromomethylase mutants

Algorithms of TEF were tuned and validated using NGS data for regenerated individuals of rice. That is, using data for previously described and biological experiment verified TEs in the paper of TIF detection algorithm of TE insertion [7]. There are two copies of *Tos17* in chromosome 7 and 10 in Nipponbare. *Tos17* are activated in cultured cells and transposed. *Tos17s* are immediately inactivated in regenerated individuals. TIF detected four and eleven *Tos17* insertions in regenerated line ttm2 (SRR556173) and ttm5 (SRR556174 and SRR556175), respectively. The result of TEF algorithm 1 (TSD method) using 5 bases of TSD length is shown in Additional file 3: Table S1 and the map of transposed positions is shown in Fig. 2. TEF algorithm 2 (Junction method) returned the same result. Positions of junction, *i.e.,* inserted positions, are determined by flanking sequence of head and tail. The distance between junctions should be the length of the TSD. Positions are selected by counting NGS reads containing junctions of head and tail, and non-insertion sequences. One or more head and tail junctions in addition to two or more non-insertion read counts in another sample is the filtering rule to detect true transpositions. High frequency count of non-insertion indicates that the target of TE transposition is a highly repetitive region. All inserted positions detected by TIF have been detected, and head and tail pair of *Tos17* also has been detected by TEF. The insertion in repetitive locus also detected as candidate positions. Detected head and tail sequences and number of TSD for each line are shown in Additional file 4: Table S2. The difference between number of detected positions in Additional file 3: Table S1 and number of TSD in Additional file 4: Table S2 is due to insertion of repetitive regions. In the regenerated cells from cell culture, the only detected active TE was *Tos17. *

The new insertions of ttm2 and ttm5 are the result of activation and insertion of *Tos17* by cell culture. Because *Tos17* is a *copia* type retrotransposon and has a long terminal repeat (LTR), head and tail sequences are detected twice within the LTR in single *Tos17* (Table S3 in Additional file 5). Size of TE can be estimated from chromosomal positions of head and tail sequences. *Tos17* on chromosome 7 and 10 are 4114 bp (26,698,889 + 20 − 26,694,795) and 4204 bp (15,419,558 + 20 − 15,415,374), respectively. Sizes of *Tos17* matched the result of the biological analysis [8]. The TEF detection performance of TE for rice regenerated lines is exactly the same as the TIF result, indicating sensitivity and precision of TEF are the same as those of TIF. In addition, TEF can detect *Tos17* transposition without sequence information of TEs which is not possible with TIF.

### Detection of transposition of *ONSEN* in *Arabidopsis thalian*a

*ONSEN (ATCOPIA78)* retrotransposon is activated by heat treatment at 37 °C for 20 h, growing on gel containing alpha-amanitin and zebularine [9]. Two fastq files (hcLine4 and hcLine31) were analyzed by junction method of TEF. The *ONSEN* element was detected with head and tail pair 5’-TGTTGAAAGTTAAACTTGAT and AAAAGAATTTTACTCTAACA-3’. Transpositions of *ONSEN* are shown in Additional file 1: Fig. S1. Number of detected transpositions of hcLine4 and hcLine31 are 32 and 9, respectively. Insertion positions and genotype are listed in Additional file 6: Table S4. Other TEs were not detected in the heat-treated plants.

### Detection of transpositions in *Oryza sativa cmt3a* mutant

Choromomethylase gene *OsCMT3a* involves DNA methylation of chromatin, and the mutation of gene affects TE transposition [10]. *OsCmt3a-1* mutants (SRR1598921, SRR1609155 and SRR1610772) are disrupted the gene by *Tos17* at chromosome 10, position 384,163 confirmed by TIF program. *OsCmt3a-2* mutants (SRR1609931, SRR1609959, and SRR1609962) are disrupted by *Tos17* at chromosome 10 position 382,197. M_4_ generation of *OsCmt3a-1* (SRR1610772) and *OsCmt3a-2* (SRR1609962) have been analyzed by the junction method (Table S5 in Additional file 7, S6 in Additional file 8 and Fig. 2). *OsCmt3a-1* and *OsCmt3a-2* mutants have new insertions of *Dasheng*, *Osr4.3* and *Osr25.4*.

### Detection of transpositions in *Arabidopsis thaliana ddm1* mutant

Mutation in a SWI2/SNF2 chromatin remodeling protein (*DDM1*) affects DNA methylation state in the genome DNA and causes activation of TEs [11, 12]. Fastq files, DRR001193 and DRR001194 are from *ddm1* mutant. They have G to A mutation (C615Y) in chromosome 5, position 26,652,152 in the TAIR10 reference genome, confirmed by the PED program [13]. Insertion points and category of TEs are listed in Additional file 9: Table S7 and Additional file 10: Table S8. New insertions of *CACTA, Hi, Evade, Ty1/copia, Ty3/gypsy* elements have been detected by junction method (Fig. S1 in Additional file 1) [14, 15]. Notably, *Evade* retrotransposon (AT5G17125) showed high frequency transposition.

### Detection of transpositions of TEs in core collections of rice

**Fig. 3:**
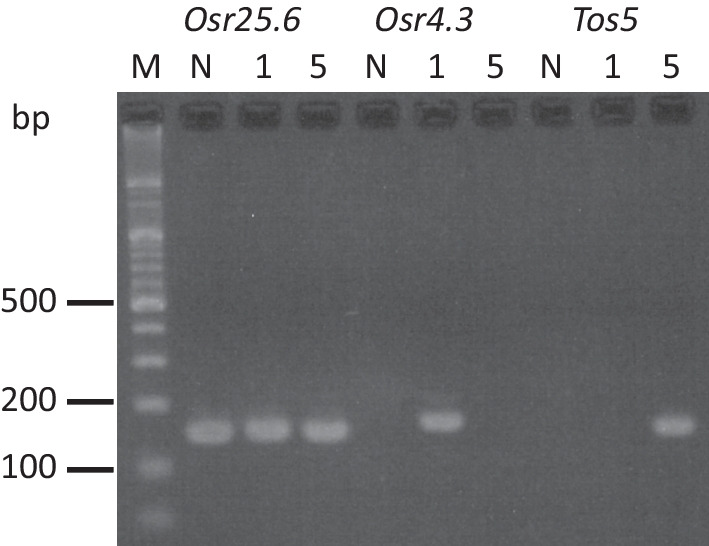
Detection of specific transposition between JRC cultivars. M: 100 bp ladder marker, N: Nipponbare, 1: JRC01, 5: JRC05. Reactions of *Osr25.6* are positive control. Electropherogram shows fragments as a result of transposition of *Osr4.3* in JRC01 and *Tos5* in JRC05, respectively

To detect active TE in rice, rice core collections of Japanese landraces JRC01 (DRR240814 in DRP006572, cv. Gaisen Mochi) and JRC05 (DRR240817, cv. Yamada Bake) have been analyzed [16]. A total of 44 types of TE head and tail sequences have been detected. List of inserted positions and genotype is shown in Additional file 11: Table S9. Category of TEs and their TSD counts in are listed in Additional file 12: Table S10. Figure 2 shows examples of TE transpositions between JRC01 and JRC05 detected by the junction method. *Osr4.3* (5’-TGTTAGAATTTCAATGGTTA and AGCCAAGACTTATTCCTACA-3’) insertions have been detected on chromosome 4 and 8 in JRC01 [17]. *Tos5/Osr13/Houba* (5’-TGTTGGAATTAATGAATGGG and ATATGCCCATAATCTCAACA-3’) insertions have been detected on chromosome 1, 3, 6, 7, 8, 12 in JRC05 [18]. Polymerase chain reaction with primer pairs across junctions (chromosome 4 position 7,892,312 for *Osr4.3*, and chromosome 8 position 6,122,658 for *Tos5*) have shown specific amplification with JRC01 and JRC05 DNA, respectively (Fig. 3).　TE head and tail sequences detected by junction method from each JRC line and Nipponbare short reads (DRR086972) are listed in Additional file 13: Table S11. A total of 328 types of TE head and tail sequences have been detected. Especially, 71 types start with TG and end with CA.

### Detection of transpositions in *Drosophila melanogaster*

In the TIF paper, we demonstrated detection of *P* element transposition. Using same short read sequences (SRR823377 and SRR823382), the pair of head and tail sequence of *P* element has been detected by TSD method of TEF. Head and tail sequences of *P* element have been detected with head and tail pair 5’-CATGATGAAATAACATAAGG and CCTTATGTTATTTCATCATG-3’. Detection of *P* element failed by junction method, because the *P* element sequence is absent from reference genome. In addition, head and tail (5’-CAGAGAACTGCAAGGGTGGC and TGGCGGGCTGCAGTTCTCTG-3’) of *Hobo* MITE element as results of transposition have been detected by both TSD and junction methods [19]. Figure S2 in Additional file 2 shows distribution of TE transposition. Transpositions of transposon *FB4* (5’-AGCTCAAAGAAGCTGGGGTC and GACCCCAGCTTCTTTGAGCT-3’) have been detected 356 and 60 positions in SRR823377 and SRR823382, respectively (containing repetitive regions) [20]. Transposition of retrotransposon *roo* (5’-TGTAAAATCCCAAATAAGAA and TTCGTGTTCATGTGTGAACA-3’) also detected with high frequency [21]. Overall, 29 kinds of TE end sequence have been detected by the junction method in SRR823377 and SRR823382. By the junction method, the largest size of detected TSDs was 10 bases (Table S12 in Additional file 14 and S13 in Additional file 15).

### Read depth and coverage of TE detection by TEF

**Fig. 4:**
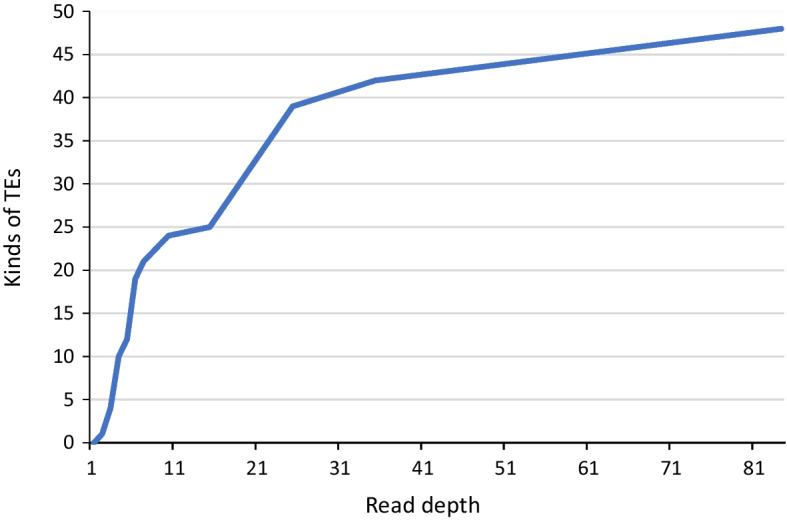
TE detection efficiency in different read depth for genome size. Sequences from the *Drosophila melanogaster* (SRR823377 and SRR823382) are selected by read depth. Each set of selected reads has been analyzed by TEF and kinds of detected TEs are plotted

Numbers of detected TEs from different read depth data of the *Drosophila melanogaster* (SRR823377 and SRR823382) by algorithm 2 (Junction method) are plotted on Fig. 4. Depth of total sequence was 84-fold of the reference genome sequence. Number of TEs is double counted with complementary sequence. TE without complementary sequence is detected at first in the low depth reads and the complementary TE is detected in the higher depth reads. At least 40-fold (20-fold from both samples) reads of genome size is required for almost complete detection of TEs by TEF.

### Run time of TEF analysis

Run time of real CPU usage for *Drosophila melanogaster* with total 115,102,984 reads (SRR823377 and SRR823382, 11.5 Giga base, 84-fold reference genome) for junction and following TSD methods were 10.7 h, and 8.8 h respectively, using Intel Core i9-10,900 CPU @ 2.80 GHz, 16 GB memory, 2 TB M.2 NVMe SSD and Ubuntu 20.04 LTS. For the analysis of rice with total 587,020,986 reads (ttm2 and ttm5, 58.7 Giga base, 157-fold reference genome), junction and following TSD methods were 73 h, and 71 h respectively. The long run time is due to the step of saving sorted *k-*mer sequences on disk.

### Comparison with other TE detection tool

Other TE detection tools, *e.g.,* TEMP2, PopoolationTE2, and TEFLoN, detect TEs using bwa for mapping of short reads to the reference genome sequence [22–24]. These programs require pair-ends short reads to detect insertion junctions by one of pair mapped to reference genome and the other mapped to a set of TE reference sequences. These detection tools require a set of TE reference sequence information. To compare performances of TEF and TEFLoN, NGS data from two sublines of *Drosophila melanogaster,* 33–45 (SRR866312) and 33–27 (SRR866314), are analyzed by TEF (Table S14 in Additional file 16 and S15 in Additional file 17). Unfortunately, due to different version of reference genome, transposed positions could not be compared correctly. Transposition of *P, hobo, hopper,* and *roo* elements were detected by both TEF and TEFLoN. TEF detects transposition of a *Ty3/gypsy* type retrotransposon which could not detected by TEFLoN. However, TEs detected by TEFLoN in single subline, *e.g., G* and *Rt1* Non-LTR retrotransposons could not be detected by TEF.

## Discussion

Focusing of TSD, two algorithms to find active TEs were developed. Algorithm 1 (TSD method) is based on pairing TSDs. Algorithm 2 (Junction method) is based on mapping of flanking sequences of head and tail of estimated TEs. Advantages and limitations of TEF are listed in Table 2. Because TEF uses only information of junction and TSD sequences as a result of transposition, similarity search against TE reference sequences is not required to select TE candidates. Due to the requirement for TSD information, TEF cannot detect TEs which do not make TSD, *e.g.,* non-LTR retrotransposons and Helitron. Many TE detection tools require pair ends short reads to detect junctions with one end mapped to reference and the other mapped to the TE reference. Because TEF detects junction within a read, single end reads can be analyzed. TEF (Algorithm 1) detects transpositions by different combination of TSDs between two samples, TEF cannot detect TEs from one sample. However, TE transposition in one sample can be detected by algorithm 2 (Junction method). Due to dependency of sequence difference between samples, TEF detects ‘transposed’ TEs and does not detect already existing TEs at same genomic positions in both samples. Algorithm 1 (TSD method) detects head and tail candidate of TEs by pairing TSD sequences. Reference genome sequence is not required for algorithm 1. That is, active TEs can be detected from species which do not have reference genome sequence. In addition, algorithm 1 (TSD method) can detect TEs which are absent from the reference genome sequence. However, algorithm 2 (Junction method) requires the reference genome sequence, because mapping and junction detection on the reference genome sequence is the principle of the algorithm. Although TEF can not detect TE excision, another algorithm, PED, can detect long deletions [13]. The combination of TEF and PED analyses could be an effective way for the study of TE dynamics.

**Table 2:** Advantage and limitations of TEF

Advantage and limitations	Algorithm 1 TSD method	Algorithm 2 Junction method
Detect TE transposition without TE reference sequence	Yes	Yes
Detect TE transposition which does not make TSD during transposition or with broken TSD	No	No
Analyze single-end reads	Yes	Yes
Detect TE transposed in one sample	No	Yes
Detect only transposed TEs	Yes	Yes
Detect TE transposition without reference sequence	Yes	No
Detect TE transposition not in reference sequence	Yes	No
Always detect TE transposition from different combinations of samples	No	No
Detect TE excision	No	No

Both algorithms detect TEs by comparison sequences between two samples. TEs detected by TEF vary with the combination of samples. TEF use with different combinations of samples may improve detection accuracy. All combinations of TSD and head–tail pairs for TE candidates are hard to keep in-computer memory. Instead of the in-memory analysis, TEF program saves temporary data into disk space. This enables to detect of all active TEs by comparing all nucleotide sequences from two samples, although with relatively slow calculation speed. The bottleneck of calculation is the sorting of *k*-mer sequences from fastq sequence. Because the data set of sorted *k-*mer sequences saved on the disk is reusable, additional TEF analysis can be performed without this bottleneck.

Using fastq files from regenerated individuals of rice which have inserted sites of retrotransposon *Tos17* already identified with the biological experiment, both algorithms detected the pair of head and tail sequences of *Tos17* and their TSDs and insertion positions. The result indicates that accuracy of TEF for detection of TE transposition is similar to that previously described in the TIF paper. Transpositions of heat induced retrotransposon *ONSEN* in *Arabidopsis thaliana* are also detected by TEF. Comparison of TEF and TEFLoN indicates that TEF fails TEs transposed in only one sample and Non-LTR retrotransposons which do not make TSD. On the other hand, TEF detects transposition of a *Ty3/gypsy* type retrotransposon. This TE could not be detected by TEFLoN.

It is known that activation of TE elements is triggered by change of methylation state of genome [11]. TEF analysis of two mutants of *OsCMT3a* gene which are disrupted by insertion of *Tos17* at different positions detects new transpositions of *Dasheng* and *Osr4.3* and *Osr25.4*. On the other hand, *Arabidopsis ddm1* mutant has a mutation in SWI2/SNF2 chromatin remodeling protein which involves DNA methylation. Transpositions of *CACTA* transposon in the *ddm1* mutant has been detected by TEF program and confirms previous reports [12]. In addition, TEF detects transpositions of other TEs, *Hi,* AT5G17125 (*Evade*), AT5G44925 and AT2G13940 in the *ddm1* mutant. These elements might be also activated with the change of chromatin remodeling state by the *ddm1* mutation.

*Japonica* rice core collections (JRC) are selected from rice genetic resources in NARO and used for genetic analysis of diversity. More than 300 pairs of TE end candidates have been detected among JRC collections by TEF program. Notably, 71 of candidates have ‘TG’ and ‘CA’ consensus of rice retrotransposon at the ends of TE head and tail sequences (Table S11 in Additional file 13). TEF detects transpositions of *Osr4.3* and *Tos5* in JRC01 and JRC05. Detected transpositions are specific events in JRC01 and TRC05 (Fig. 3), indicating TEF method is applicable for the analysis of TE dynamics among cultivars.

Detected head-tail pair sequence can be used as a unique identifier of TE for whole organisms. For example, *Tos5* has alternative names *Osr13* and *Houba.* The multiple name and IDs of TEs is an obstacle to understanding for TE dynamics among species. If head-tail sequence, such as TGTTGGAATTAATGAATGGG-ATATGCCCATAATCTCAACA for *Tos5/Osr13/Houba,* is used for the unique identifier, TE distribution among species or genus can be explained more clearly. It should be considered that aged TSDs sometimes mutated. TEF cannot detect transposition with mutated TSDs. Although conserved head, tail, and TSD sequences are required, TE distribution among species detected by TEF with the unique IDs will contribute to know the relation between species and TE evolutions.

Short read sequences from SRR823377 and SRR823382 are from recombinant inbred lines of *Drosophila melanogaster* WE70 and *yw* cross. Both fly lines show extreme cardiac phenotype [25]. Sequence of *Drosophila P* element is not present in the reference genome sequence. Due to the absence of *P* element sequence, junction of transposed *P* element is not able to be detected by algorithm 2 (Junction method). Fortunately, algorithm 1 (TSD method) detects head and tail pair of TE even in the absence of the reference sequence. Other than *P* elements, transpositions of *hobo* element were also detected. By the junction method, more than 30 kinds of TEs have been detected. The large number transpositions of TEs causes high frequency phenotypic change of crossed lines. Activation of TEs may be due to the crossing of WE70 and *yw*. By the crossing of two lines with different chromosomal state, new interactions among transposase of TE and chromosomal DNA and/or proteins may have occurred. The difficulty of mapping the extreme cardiac phenotype into a single locus may be due to TE transpositions which cannot be detected by standard analysis flow for NGS sequences.

TEF reveals many TE transpositions in low methylation backgrounds such as *ddm1* of *Arabidopsis thaliana* and *OsCmt3a* of *Oryza sativa* mutants*.* In addition, transposition events were also detected in rice cultivars and recombinant inbred lines of *Drosophila*. The results bring to mind the hypothesis that an imbalance of methylation and/or chromatin state by crossing-over causes an activation of sleeping transposons.

For a long time, the enormous amounts of noncoding or repetitive sequence in the genome have been hard to analyze. For the insertion in repetitive regions, TEF will output candidates of TE transposed position. Thus we will know if the TE is active and transposed into repetitive area. The total transposed number of TE can be estimated by counting the unique number of TE flanking sequences (Table S11 in Additional file 13).

If the base(s) outside of TSD is/are the same as the first or last base(s) of TE, both methods return multiple patterns of transposition. For example, *P* element was transposed from 21,836 to 21,844 in chromosome 2L (Table S12 in Additional file 14). The TE is detected three times with different positions and different length of TSD, due to the same base between the end of TE and TSD. Because TEF detects TEs with only TSD information, the multiple counting is unavoidable and should be considered as an error source. Implementation of filtering rule for multiple counting will be the next challenge for TEF update. In addition, TEF does not detect already existing intact TEs at the same genomic positions in both samples. TEF detects only ‘Transposed’ TEs between samples. Although this makes it difficult for direct comparison of sensitivity and precision to other TE detection programs, TEF succeeded in detecting TE transpositions which could not be found by other detection programs.

For short TSDs, 3 bp or less, TEF with algorithm 1 (TSD method) does not work well due to limitation of the types of TSD sequence. In the case of two insertions located in different positions on the genome having the same TSD sequence, exact detection of transposition by the TSD method will be difficult. However, using the junction method, flanking sequence of TE will be mapped to each insertion position and transpositions can be mapped to each position. In addition, algorithm 1 (TSD method) can detect TE transposition without a reference genome sequence. The distribution of detected TEs among species as well as cultivars will be useful information for understanding of genetic lineage and breeding history.

## Conclusion

Until now, it has been difficult to detect TE transposition by the standard NGS analysis flow, because TEs themselves are already on the chromosome with multiple copies. The new tool TEF, focusing on TSD created by the TE transposition, is an effective new method to explore the unknown genome areas, especially for TE dynamics. The method is applicable to both plant and animal genomes.

## Methods

A set of JRC lines was obtained from the Genetic Resources Center, NARO. Fastq sequences described in the result section have been downloaded from the Sequence Read Archive (SRA) / NCBI. Fastq files for detection of *ONSEN* elements were obtained from https://zenodo.org/record/5052019. Reference genome sequence, IRGSP1.0 for rice, TAIR10 for *Arabidopsis*, and dmel_r6.26 for *Drosophila melanogaster* are downloaded from https://rapdb.dna.affrc.go.jp/download/archive/irgsp1/IRGSP-1.0_genome.fasta.gz, ftp://ftp.ensemblgenomes.org/pub/plants/release-41/fasta/Arabidopsis_thaliana/dna/Arabidopsis_thaliana.TAIR10.dna.toplevel.fa.gz, ftp://ftp.flybase.net/genomes/Drosophila_melanogaster/dmel_r6.26_FB2019_01/fasta/dmel-all-chromosome-r6.26.fasta.gz, respectively [26–28]. Fastq files were obtained from NCBI SRA https://www.ncbi.nlm.nih.gov/sra with accession number described in result section.

### Implementation of algorithms

Methods of excluding PCR bias in short-reads, slicing and sorting of *k-*mer, and mapping on reference genome sequence were described previously [13]. TSD and junction methods have been implemented to the perl script, tef.pl. The tef.pl runs on Unix (Linux, MacOS and FreeBSD) operating system. At the first run of tef.pl, tef.pl will make dataset of reference genome. The tef.pl will output a config file in the directory of reference. The config file is a list of chromosome names from the fasta data of reference sequence. If chromosome (contig) name in the config file is edited to NOP, the corresponding sequence in the fasta file will be ignored. The second run after the edit of config file, tif.pl makes data set of reference sequence and then detects transposition events. Options are specified with one argument separated by comma. For example, to detect TE transpositions between rice ttm2 and ttm5 mutant,

perl tef.pl a = ttm2,b = ttm5,ref = IRGSP1.0

Default algorithm is the junction method. If TSD size is specified, tef.pl runs with the TSD method.

perl tef.pl a = ttm2,b = ttm5,ref = IRGSP1.0,tsd_size = 5

### Confirmation of specific transpositions by biological analysis

DNAs from JRC lines have been purified from leaves of mature plants by CTAB method [29]. DNAs from JRC01 and JRC05 (10 ng) are amplified by GoTaq (Promega) and 0.2 μM primers with 35 thermal cycles at 94 °C for 15 s, 60 °C for 60 s, and 72 °C for 30 s. Reaction mixtures were electrophoresed with 3% agarose gel in Tris-Borate-EDTA buffer. Gel was stained with 0.5 μg/ml ethidium bromide and then products were visualized with 254 nm ultraviolet light (Fig. 3). Primer sequences for specific transposition of *Osr4.3* at 7,892,312 on chromosome 4 in JRC01 (DRR240814) are TTGAACAGTACGCAGGTGAC (PB1343) and TCTTTGGGAGGACATGGCTT (PB1344). To detect the specific transposition of *Tos5* at position 6,122,658 on chromosome 8 in JRC05 (DRR240817), primers with nucleotide sequences TGGTTTTGGGGCTGAACATG (PB1347) and TCTTCGAGTGGGCTAAGACC (PB1348) are used. For the reaction control, primers with nucleotide sequences ATGCACAAGAAGAATGACTGC (PB1345) and ACCCATACACTTTCCAAGCTTG (PB1346) to detect *Osr25.6* insertion at 6,122,658 on chromosome 8 in Nipponbare, JRC01 and JRC05 are used.

## Supplementary Information

**Additional file 1. Figure S1.** TE transpositions in *Arabidopsis thaliana*. Heat treated lines hc31 and hc4 show transposition of *ONSEN*. DRR001193 and DRR00194 are *ddm1* mutants. Transpositions of *CACTA, Evade* and AT2G13940 are shown.

**Additional file 2. Figure S2.** TE transpositions in RILs of *Drosophila melanogaster*. Insertion positions of *P, FB4, Hobo* and *roo* elements detected by TEF are indicated with vertical lines in red for accession SRR82377 from RIL46 (WE70/*yw*) and blue for SRR82382 from RIL80 (WE70/*yw*).

**Additional file 3. Table S1.** Detected head and tail sequence of TE and their inserted positions on the reference sequence by TSD method.

**Additional file 4. Table S2.** Detected head and tail sequence of TE and kinds of TSD in ttm2 and ttm5 by TSD method.

**Additional file 5. Table S3.** Detected head and tail sequence original TE in the ttm2 genome by TSD method.

**Additional file 6. Table S4.** Detected head and tail sequence of TE and their inserted positions on the reference sequence for hc4 and hc31 by TSD method.

**Additional file 7. Table S5.** Detected head and tail sequence of TE and their inserted positions on the reference sequence for SRR1609962 and SRR1610772 by Junction method.

**Additional file 8. Table S6.** Detected head and tail sequence of TE and kinds of TSD in *OsCmt3a-1* (SRR1610772) and *OsCmt3a-2* (SRR1609962) by Junction method.

**Additional file 9. Table S7.** Detected head and tail sequence of TE and their inserted positions on the reference sequence for DRR001193 and DRR0011934 by Junction method.

**Additional file 10. Table S8.** Detected head and tail sequence of TE and kinds of TSD in DRR001193 and DRR001194 by Junction method.

**Additional file 11. Table S9.** Detected head and tail sequence of TE and their inserted positions on the reference sequence for JRC01 and JRC05 by Junction method.

**Additional file 12. Table S10.** Detected head and tail sequence of TE and kinds of TSD in JRC01 and JRC05 by Junction method.

**Additional file 13. Table S11.** Head and tail sequences of TE element among JRC lines detected by junction method. Head, tail and flanking sequences are detected by comparison between JRC lines and Nipponbare short reads. Line names of TE insertion with same flanking sequences are listed.

**Additional file 14. Table S12.** Detected head and tail sequence of TE and their inserted positions on the reference sequence for SRR823377 and SRR823382 by Junction and TSD methods.

**Additional file 15. Table S13.** Detected head and tail sequence of TE and kinds of TSD in SRR823377 and SRR823382.

**Additional file 16. Table S14.** Detected head and tail sequence of TE and their inserted positions on the reference sequence for SRR866312 and SRR866314 by Junction and TSD methods.

**Additional file 17. Table S15.** Detected head and tail sequence of TE and kinds of TSD in SRR866312 and SRR866314.

## Data Availability

The TEF software is available at: https://github.com/akiomiyao/tef Operating system: Unix (Linux, MacOS and FreeBSD) Programming language: perl License: Free of use for academics. For non-academics, license by NARO is required.

## Abbreviations

NGS: Next-generation sequence
NOP: No operation
PED: Polymorphic edge detection
RIL: Recombinant inbred line
TEF: Transposable element finder
TIF: Transposon insertion finder
TSD: Target site duplication
