# Supplementary material for: Transposable element finder (TEF): finding active transposable elements from next generation sequencing data
Source: BMC Bioinformatics. 2022 Nov 22;23:500. doi: 10.1186/s12859-022-05011-3 (PMC9682801; doi:10.1186/s12859-022-05011-3)
Supplement: Supplementary file 1 — Additional file 1. Figure S1. TE transpositions in Arabidopsis thaliana. Heat treated lines hc31 and hc4 show transposition of ONSEN. DRR001193 and DRR00194 are ddm1 mutants. Transpositions of CACTA, Evade and AT2G13940 are shown. [file 12859_2022_5011_MOESM1_ESM.pptx]

## Slide 1
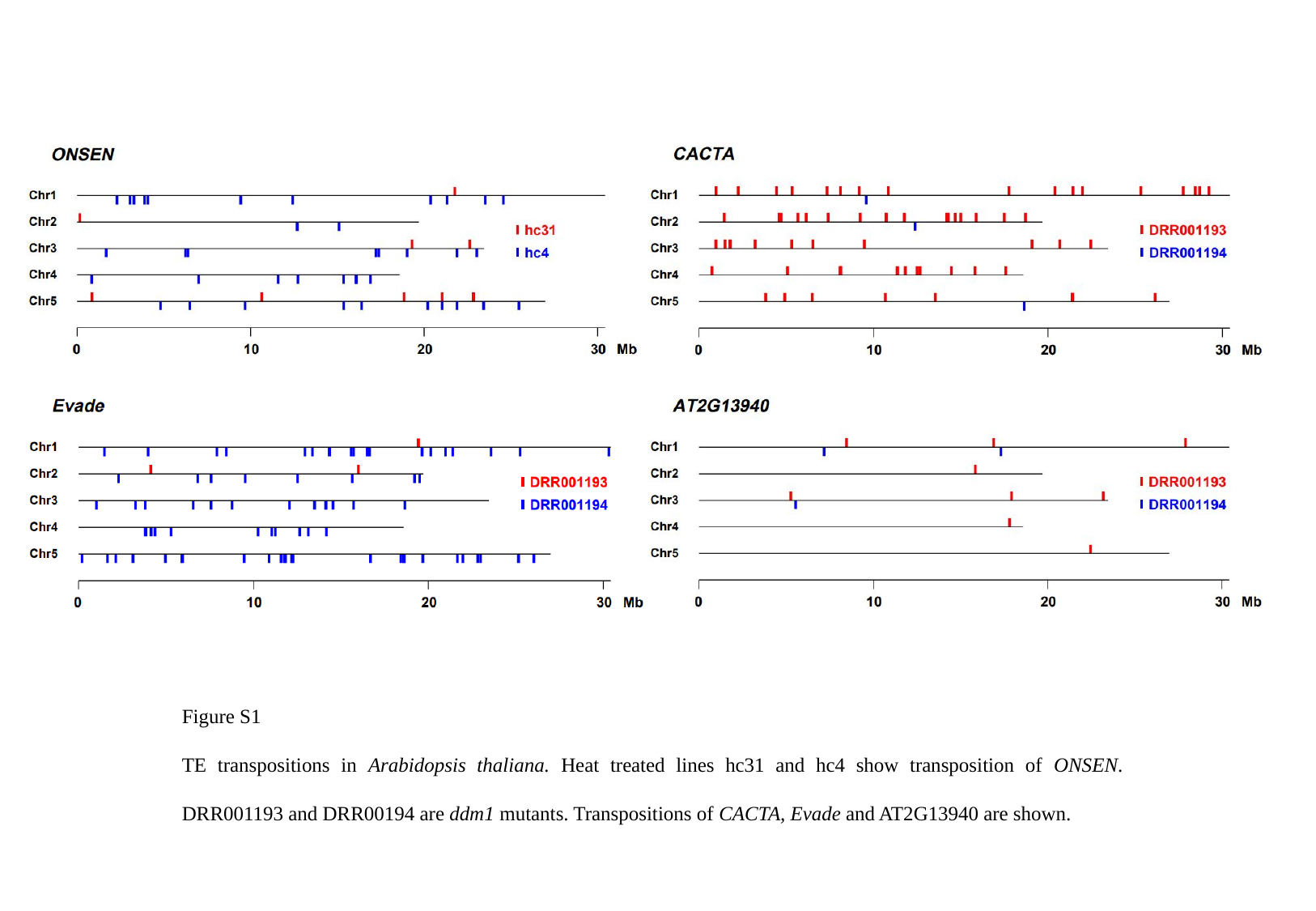

Figure S1
TE transpositions in Arabidopsis thaliana. Heat treated lines hc31 and hc4 show transposition of ONSEN. DRR001193 and DRR00194 are ddm1 mutants. Transpositions of CACTA, Evade and AT2G13940 are shown.
