# Supplementary material for: Transposable element finder (TEF): finding active transposable elements from next generation sequencing data
Source: BMC Bioinformatics. 2022 Nov 22;23:500. doi: 10.1186/s12859-022-05011-3 (PMC9682801; doi:10.1186/s12859-022-05011-3)
Supplement: Supplementary file 2 — Additional file 2. Figure S2. TE transpositions in RILs of Drosophila melanogaster. Insertion positions of P, FB4, Hobo and roo elements detected by TEF are indicated with vertical lines in red for accession SRR82377 from RIL46 (WE70/yw) and blue for SRR82382 from RIL80 (WE70/yw). [file 12859_2022_5011_MOESM2_ESM.pptx]

## Slide 1
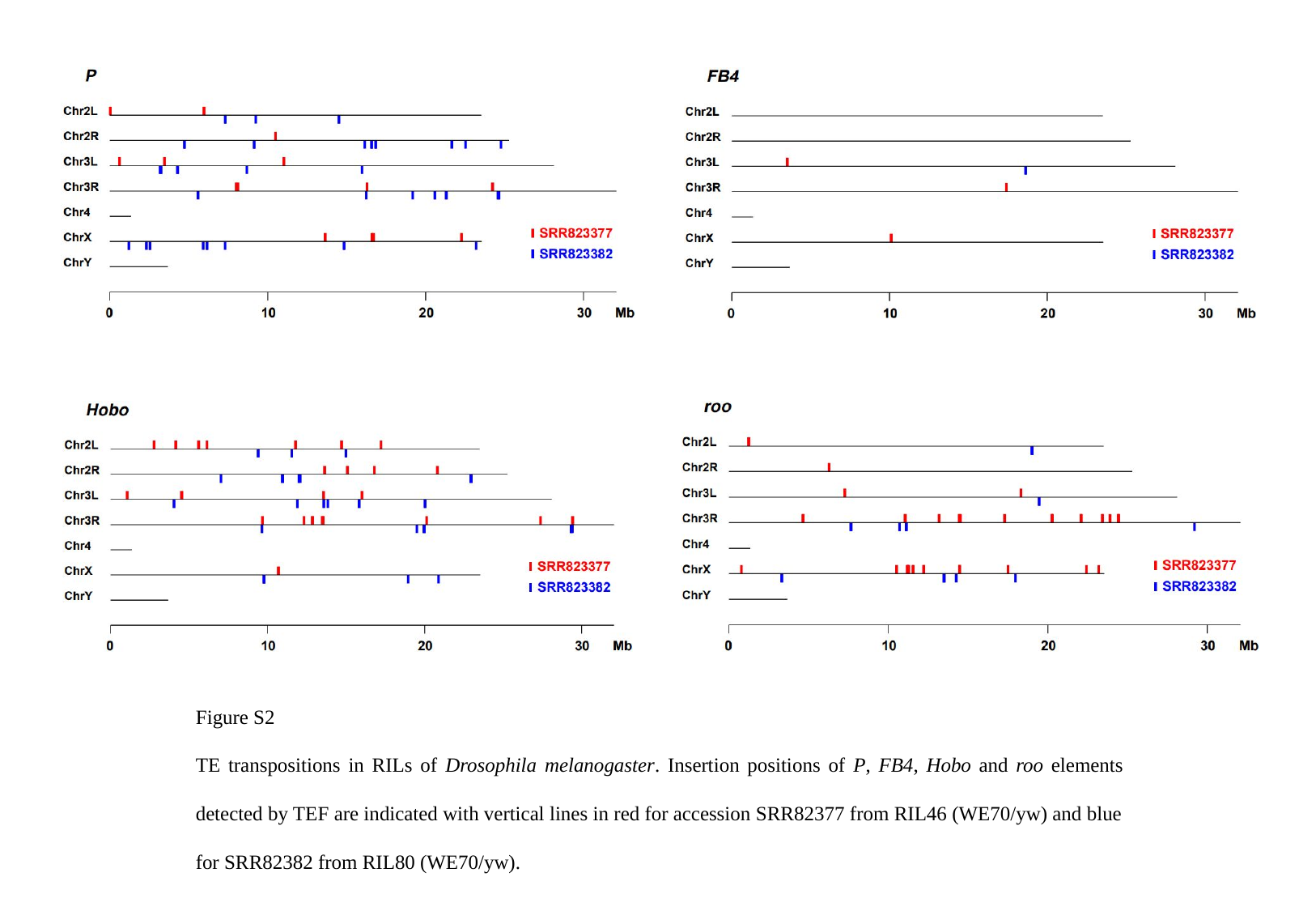

Figure S2
TE transpositions in RILs of Drosophila melanogaster. Insertion positions of P, FB4, Hobo and roo elements detected by TEF are indicated with vertical lines in red for accession SRR82377 from RIL46 (WE70/yw) and blue for SRR82382 from RIL80 (WE70/yw).
